# Temperature-driven coordination of circadian transcriptional regulation

**DOI:** 10.1371/journal.pcbi.1012029

**Published:** 2024-04-22

**Authors:** Bingxian Xu, Dae-Sung Hwangbo, Sumit Saurabh, Clark Rosensweig, Ravi Allada, William L. Kath, Rosemary Braun

**Affiliations:** 1 Department of Molecular Biosciences, Northwestern University, Evanston, Illinois, United States of America; 2 NSF-Simons Center for Quantitative Biology, Northwestern University, Evanston, Illinois, United States of America; 3 Department of Biology, University of Louisville, Louisville, Kentucky, United States of America; 4 Department of Neurobiology, Northwestern University, Evanston, Illinois, United States of America; 5 Department of Biology, Loyola University, Chicago, Illinois, United States of America; 6 Michigan Neuroscience Institute, University of Michigan, Ann Arbor, Michigan, United States of America; 7 Department of Anesthesiology, University of Michigan, Ann Arbor, Michigan, United States of America; 8 Northwestern Institute on Complex Systems, Northwestern University, Evanston, Illinois, United States of America; 9 Department of Engineering Sciences and Applied Mathematics, Northwestern University, Evanston, Illinois, United States of America; 10 Department of Physics and Astronomy, Northwestern University, Evanston, Illinois, United States of America; 11 Santa Fe Institute, Santa Fe, New Mexico, United States of America; Universitat zu Koln, GERMANY

## Abstract

The circadian clock is an evolutionarily-conserved molecular oscillator that enables species to anticipate rhythmic changes in their environment. At a molecular level, the core clock genes induce circadian oscillations in thousands of genes in a tissue–specific manner, orchestrating myriad biological processes. While previous studies have investigated how the core clock circuit responds to environmental perturbations such as temperature, the downstream effects of such perturbations on circadian regulation remain poorly understood. By analyzing bulk-RNA sequencing of *Drosophila* fat bodies harvested from flies subjected to different environmental conditions, we demonstrate a highly condition-specific circadian transcriptome: genes are cycling in a temperature-specific manner, and the distributions of their phases also differ between the two conditions. Further employing a reference-based gene regulatory network (Reactome), we find evidence of increased gene-gene coordination at low temperatures and synchronization of rhythmic genes that are network neighbors. We report that the phase differences between cycling genes increase as a function of geodesic distance in the low temperature condition, suggesting increased coordination of cycling on the gene regulatory network. Our results suggest a potential mechanism whereby the circadian clock mediates the fly’s response to seasonal changes in temperature.

## Author summary

The circadian clock enables organisms to anticipate and adapt to changes in their environment. While behavioral changes have been observed in *Drosophila melanogaster* subjected to low temperatures, little is known regarding how these changes are enacted at a molecular level. By conducting bulk RNA sequencing from fruit flies, we observe that genome-wide circadian oscillation patterns are temperature dependent. Intriguingly, we find that morning and evening peaks of transcriptomic activity shift closer together, consistent with anticipation of a shorter photoperiod in cooler winter weather. We further find that the low-temperature dynamics are highly coordinated with respect to a reference-based gene regulatory network. Our findings provide insights into the mechanisms by which flies adapt to environmental temperature changes.

## Introduction

A circadian clock is present in organisms from fungi to insects to mammals, where it drives the regulation of processes such as the rest/activity cycle, spore production, and metabolism [1]. In general, the circadian clock can be defined as a robust 24-hour self-sustaining oscillator that can be entrained by environmental cues, such as light. The molecular basis of the core clock circuit has been elucidated with great detail [1–6]. In *Drosophila*, the CLOCK/CYCLE (CLK/CYC) heterodimer binds to the E-box to activate gene expression of period (*per*) and timeless (*tim*). PER and TIM proteins dimerize in the cytoplasm and translocate to the nucleus, with a time constant of approximately 4 hours, to inhibit the DNA binding activity of CLK/CYC [2]. This transcription-translation feedback loop oscillates with an approximate 24-hour period. In turn, it drives the expression levels of hundreds of downstream genes.

Recent advances in high throughput sequencing have made it possible to identify genes under circadian control. In a study conducted by Zhang et al. [7], where they sampled 12 different mouse organs every two hours for two days using microarray, it was reported that nearly 40% of all genes showed rhythmic behavior in at least one of the twelve organs that were studied. In addition, they observed little overlap of cycling transcripts between all organ pairs, suggesting that the circadian regulation is highly tissue–specific. Nevertheless, while each organ has a largely unique set of circadian genes, their phase distribution showed much less diversity, generally peaking or dipping synchronously ∼8 hours after lights–off (in a 12:12 light:dark environment).

A hallmark of the circadian clock is the insensitivity of its endogenous period to temperature, a phenomenon known as “temperature compensation” [6]. Such temperature stability is crucial to the correct functioning of the clock, and it has been observed in every species with a circadian rhythm, including bacteria [8], insects [9], and mammals [10]. This phenomenon is surprising, given the Arrhenius dependence of reaction rates on temperature [11], and it is clear that this property must be an emergent feature of interaction networks with temperature-dependent reactions that oppose each other [12]. However, the specific molecular mechanisms conferring this property are still not fully elucidated. Studies have identified transcriptional, post-transcriptional, translational, and protein–level interactions as possible contributors, including temperature–dependent transcription [13–16], gene-specific thermosensitive alternative splicing [3, 17, 18], thermosensitive polyadenylation [19], thermally–regulated translation [20–22], counterbalanced enzymatic reaction rates [23], and temperature–sensitive protein dimerization [24] and ubiquination [25].

Yet while the *period* of the clock remains constant across a wide range of temperatures, the *phase* of the clock has been observed to be temperature sensitive. For example, behavioral studies using *Drosophila* Activity Monitoring (DAM) systems have demonstrated that under normal light–dark cycles, flies show gradual increase in activity just before the light–dark/dark–light transitions, a phenomenon known as evening/morning anticipation [2]. At lower temperatures, the activity peaks show decreased separation [3, 26], with an earlier onset of evening anticipation that may reflect an adaptation for the shortened daylight hours in winter [3, 26].

While this temperature-dependent behavioral pattern has been observed in *Drosophila*, comparatively little is known about how downstream transcriptome–wide circadian activity changes in face of temperature perturbations. Early work reported that the transcriptome was modified globally by a cyclic temperature perturbation, which acted as a driver of circadian oscillation [14]. More recently, it was reported that flies subjected to different environmental temperatures under the light–dark cycle exhibited thermosensitive alternative splicing, affecting locomotor activities [18]. Taken together, these works provide evidence that temperature could have a global effect on the circadian transcriptome. However, both aforementioned studies had a sampling frequency of four hours, which hinders the detection of cycling genes and the estimation of their associated phases [27].

To further understand how temperature impacts the circadian transcriptome, we conducted genome-wide studies of circadian gene expression in fruit flies subjected to a 25°C or 18°C environment by performing bulk RNA sequencing on fat body samples with a sampling frequency of two hours. The fat body in *Drosophila* is a major endocrine organ responsible for metabolism and energy storage [28]. It has been observed that genes cycle with a 24-hour period in the fat body, with consequences for metabolism [29]. However, it is not known whether a prolonged temperature change can induce phase shifts or initiate circadian oscillation of “flat” genes. By conducting bulk RNA-sequencing, we show that both the identity and phases of cycling genes change in a temperature–dependent manner. In addition, we report a significant increase at lower temperatures in the phase synchronization of oscillating genes that are close to one another on the Reactome *Drosophila* gene regulatory network, suggesting that transcriptomic coordination may be enhanced at lower temperatures.

## Results

### Circadian transcriptional profiling overview

**Fig 1:**
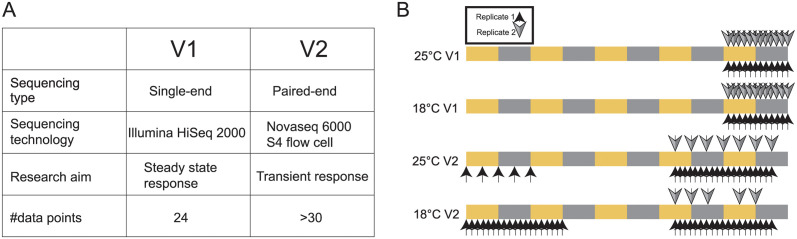
Experimental design. (A) Method and technologies used for the V1 and V2 experiments. (B) Sampling scheme of the two experiments and environmental conditions. Arrows above and below the colored bars indicate samples and replicates. The colored bars depict the light (yellow) and dark (grey) periods.

To study how the circadian rhythm is perturbed by temperature, we conducted two separate studies, henceforth termed V1 and V2. Experimental details may be found in *Materials and Methods*; we provide an overview here. Briefly, in both experiments flies were raised at 25°C before being placed in 18°C (temperature perturbation) or kept at 25°C (control). In experiment V1, *Drosophila* fat bodies were harvested every two hours in 12:12 L:D conditions starting four days after the temperature shift (see Fig 1), providing data for a stable, established circadian transcriptome. In the V2 experiment, in addition to examining the stable circadian transcriptome during days four and five, we also collected data during the first two days to investigate transient dynamics immediately following the temperature perturbation. Analysis of the transient data is reported separately [41]; here, we focus on the dynamics after flies had equilibrated to the 18°C and 25°C conditions. These two separate experiments, V1 and V2, provide the opportunity to minimize false identification of cycling genes and ensure that our results are robust to sequencing technology and experimental artifacts, which can be a major source of error in cycling detection [27, 42].

**Fig 2:**
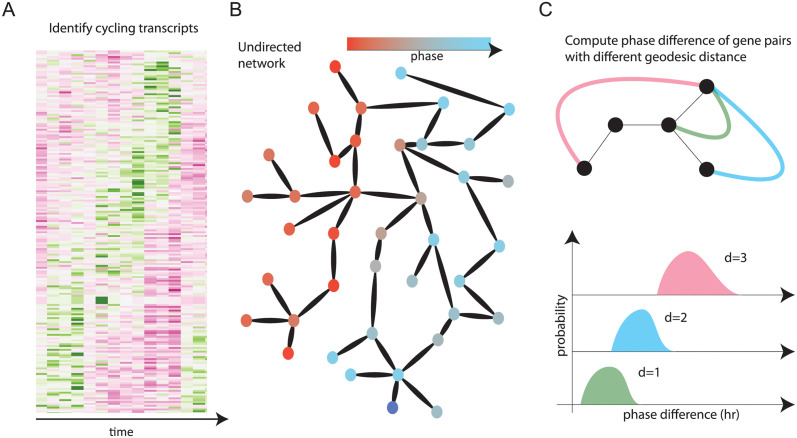
Illustration of the analysis pipeline. (A) Oscillating genes are identified under each temperature. (B) Estimated phases are mapped onto a database–derived network. (C) We search for evidence of phase organization by quantifying the distribution of phase differences between pairs of genes, as a function of their distance on the network.

**Fig 3:**
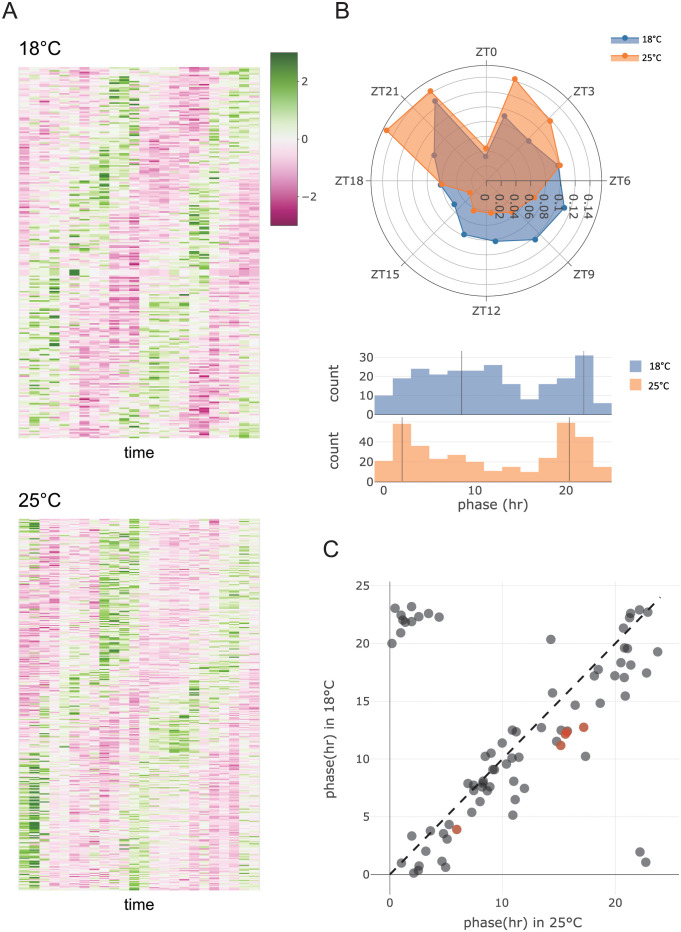
Differential cycling at 18°C vs 25°C. (A) *Z*-scored TPM (transcript per million) of detected cycling genes in the V1 experiment. (For the V2 experiment, see S2 Fig) Here, green indicates higher than average expression and pink indicates lower than average temperature for each gene. (B) Roseplot (top) and histogram (bottom) of the phase distribution for all genes cycling at either 18°C (blue) or 25°C (orange). In the histogram, the locations of each peak are estimated by fitting a two–component mixture of von Mises distributions [44, 45] and shown as black lines. (C) Estimated phases of genes that cycle at both temperatures. Red points highlight the core clock genes.

To understand how the circadian transcriptome is affected by temperature, we first identified cycling genes at 25°C and 18°C and estimated their phases (illustrated in Figs 2A and 3A). Next, we investigated whether there is evidence of increased synchronization of gene expression dynamics at different temperatures, by examining phase differences between genes in the context of putative gene regulatory networks (Fig 2B). We obtained the gene regulatory network from the Reactome database [38], an expert–curated database of experimentally–validated *Drosophila*–specific gene–gene interactions, and tested whether genes with similar phases were clustered on the graph by examining the distribution of phase differences at different graph distances (Fig 2C). Briefly, if genes close to each other on the network tend to have similar phases, we consider this evidence of phase organization. In this case, the distribution of phase differences of gene pairs will gradually increase as a function of network distance, as illustrated in Fig 2C.

### Identification of rhythmic transcripts

It has been shown that cycling detection is a challenging task, depending upon sampling resolution, cycling detection algorithm, and the dataset. To deal with these problems, we employed two strategies. First, we used harmonic regression to test for evidence of cycling, assigning the *F*–test *p*-value to each gene. We did this for both the V1 and V2 experiments, and considered a gene to be cycling only if (i) it has a harmonic regression *p* < 0.1 in *both* experiments and (ii) the estimated phases differ by less than 3 hours between the two experiments (|Δ*ϕ*| < 3*h*, Eq 3), indicating consistency between the two experiments. We also compared the estimated phases and *p*-values assigned by harmonic regression to those obtained from JTK-CYCLE [43] and observed high concordance (S1 Fig). Prior to the cycling detection step, we filtered out genes that have low expression or are rarely detected (see Materials and methods for detail), retaining 6774 genes.

The above cycling detection criteria were designed to limit the false positive rate. Under the null hypothesis of no cycling, *p*-values from harmonic regression will be uniformly distributed from zero to one. The chance of falsely obtaining *p* < 0.1 is thus 0.1 in a single experiment; obtaining *p* < 0.1 in both independent experiments V1 and V2 is thus 0.1^2^. Additionally, for false positives the estimated phase will be uniformly distributed on [0, 24) (there would be no reason to “prefer” any given phase), and thus the chance of observing |Δ*ϕ*| < 3*h* is $\frac{6}{24}$. (If the estimated phase in one experiment is *ϕ* and we require |Δ*ϕ*| < 3*h*, the other must be within *ϕ* ± 3, a 6-hour range.) All together, the probability of passing our cycling criteria by chance alone is $0.1^{2}\left(\frac{6}{24}\right)=0.0025$, so we expect 0.0025 × 6774 = 17 genes to be falsely called cycling. In our data, we identified 242 and 364 cycling genes in 18°C and 25°C respectively, of which 79 were found to be cycling at both temperatures (Fig 3A S2 Fig and S1 Table). Of these, only 17—7% and 4.7% respectively for the two temperatures—are expected to be be false discoveries (S3(A) and S3(B) Fig).

As an additional validation, we examined the dynamics of the core clock genes (*tim*, *Clk*, *vri*, *pdp*, *per*, *cry*) and observed that their estimated phases were highly concordant between the V1 and V2 experiments (S4(A) Fig). In addition, we noted a phase advance of 2–4 hours in the core clock genes at 18°C relative to 25°C (S4(B) Fig).

Taken together, this analysis suggests that genes under circadian control are temperature–specific, and that a phase advance occurs in the core clock genes at the lower temperature, potentially influencing its downstream targets.

### Functional analysis of cycling genes

To examine the function of cycling genes in each condition, we conducted an over-representation analysis on genes that cycle in 18°C, 25°C, and at both temperatures using the ReactomePA package [40] in R. The significantly over-represented pathways in 18°C and 25°C showed little overlap (S2 and S3 Tables). At 18°C we found that the pathway most strongly enriched for cycling genes is the metabolism of lipids (*q* = 0.003). In addition, many lipid-related metabolic pathways are significantly over-represented as well, such as triglyceride metabolism (*q* = 0.006), fatty acid metabolism (*q* = 0.006), carnitine metabolism (*q* = 0.02). At 25°C however, we observed only three significantly over-represented pathways: pentose phosphate pathway (*q* = 0.006), metabolism of amino acids (*q* = 0.04) and derivatives, and ABC family proteins mediated transport (*q* = 0.04). For genes that were cycling at both temperatures, we observed that the circadian clock pathway is over-represented as expected (*q* = 0.017; S4 Table). In addition, we found that metabolism of lipids to be an over-represented pathway (*q* = 0.018).

Investigating the lipid metabolism pathway more closely, we found that there were 28 and 27 cycling genes involved in the metabolism of lipids at 25°C and 18°C respectively, with only 13 genes cycling under both temperatures. In addition, for the temperature–specific cycling genes within the lipid metabolism pathway, we found that their average *p*-values at the other temperature were close to 0.4; that is, the subset of genes driving the significance of the lipid metabolism pathway are highly temperature–specific, with significant evidence of cycling at one temperature and far from significant at the other. Together, these results suggest that temperature affects lipid metabolism in the fat body, and that there are mechanisms that exercise precise control of rhythmicity even within the same pathways.

### Distribution of phases

To characterize cycling genes further, we estimated their phases at 25°C and 18°C individually by taking the circular mean of phases estimated from the V1 and V2 experiments. Interestingly, we found that the phase distributions of all detected cycling genes at both temperatures are significantly different (*p* = 0.0002, *W* = 1486, Wheeler-Watson two sample test, *df* = 605 [46, 47]) and were both bimodally distributed, peaking at ZT 2.01 and ZT 20.34 at 25°C and at ZT 8.56 and ZT 21.95 at 18°C (Fig 3B). The narrower daytime interval between the gene expression peaks observed at the lower temperature is analogous to the previous observation that morning and evening activity peaks are closer to each other at low temperature [3, 26].

To examine how rhythmic expression was perturbed by temperature, we investigated the genes that were considered cycling at both temperatures (n = 79) (i.e., the intersection of all cycling genes) and found their phases to be highly similar between temperatures, having a circular correlation [48] of 0.79 (Fig 3C). Interestingly, for genes that were considered cycling in both temperatures, we frequently observed a phase advance (Eq 2) in 18°C relative to 25°C (Fig 3C). In the V1 experiment, 61 of the 79 common cyclers show a phase advance at 18°C, with a median phase change of +1.91 hours in 18°C relative to 25°C. This is a significantly above 0 (*p* = 1.27 × 10^−6^; circular median test [49, 50]), indicating a predominant phase advance amongst the genes that cycle at both temperatures. Similar results obtain for experiment V2 (57 of 79 genes showing phase advance, with a median phase advance of +1.76 hrs in 18°C, *p* = 1.02 × 10^−4^). The concordance of the phase shifts observed in V1 and V2 is further illustrated in S5 Fig.

Interestingly, the phase shift for the common cyclers appears to be the same regardless of the peak phase of the gene; that is, among the common cyclers, we observe both the morning–peaking genes and the evening–peaking genes having a phase advance in 18°C. This implies that the differential change in the peaks of the phase distribution observed in Fig 3B are primarily attributable to temperature–specific cyclers.

In summary, we observed that environmental changes can impact the circadian transcriptome in two ways. First, circadian control of gene expression is temperature–specific, with many genes detectably cycling at only one temperature and either not oscillating, or oscillating with very small amplitude, at the other. Second, temperature impacts the phase of rhythmic genes, potentially mediated by the phase advance observed in the core clock genes.

### Network phase organization

We next examined phase organization with respect to the gene regulatory network obtained from the Reactome database [38] via the R Graphite library [36, 37]). Since the network only contained a subset of the genes, we first checked whether these were representative of all genes by testing whether the phase distribution of genes on the network differed from that of all detected cycling genes. We found that the Reactome genes did not have a significantly different phase distribution relative to all genes (18°C: *p* = 0.82, *W* = 14.53, *df* = 241; 25°C: *p* = 0.33, *W* = 124.04, *df* = 363; Wheeler-Watson two sample test [46, 47]) suggesting that these are a representative sample. We then tested whether the connectivity of a gene is predictive of its rhythmicity. Comparing the degree distributions of 18°C and 25°C cycling genes to that of all genes, we observed that these distributions were not significantly different from one another (S6(A) Fig; 18°C vs 25°C: *p* = 0.2, *D* = 0.13; 18°C vs all: *p* = 0.17, *D* = 0.11; 25°C vs all: *p* = 0.48, *D* = 0.07; Kolmogorov-Smirnov test).

We then examined whether cycling genes are co-localized on the network by computing pair-wise distances between 18°C and 25°C cycling genes separately, and comparing these distance distributions to that obtained from all genes. We found that the cycling genes at both 18°C and 25°C tend to have smaller network distances than expected from all genes on the network (S6(B) Fig; 18°C cyclers vs all: *p* = 2 × 10^−16^; 25°C cyclers vs all: *p* = 2 × 10^−16^; Wilcoxon rank sum test). This finding implies localization of cycling genes with respect to the graph.

**Fig 4:**
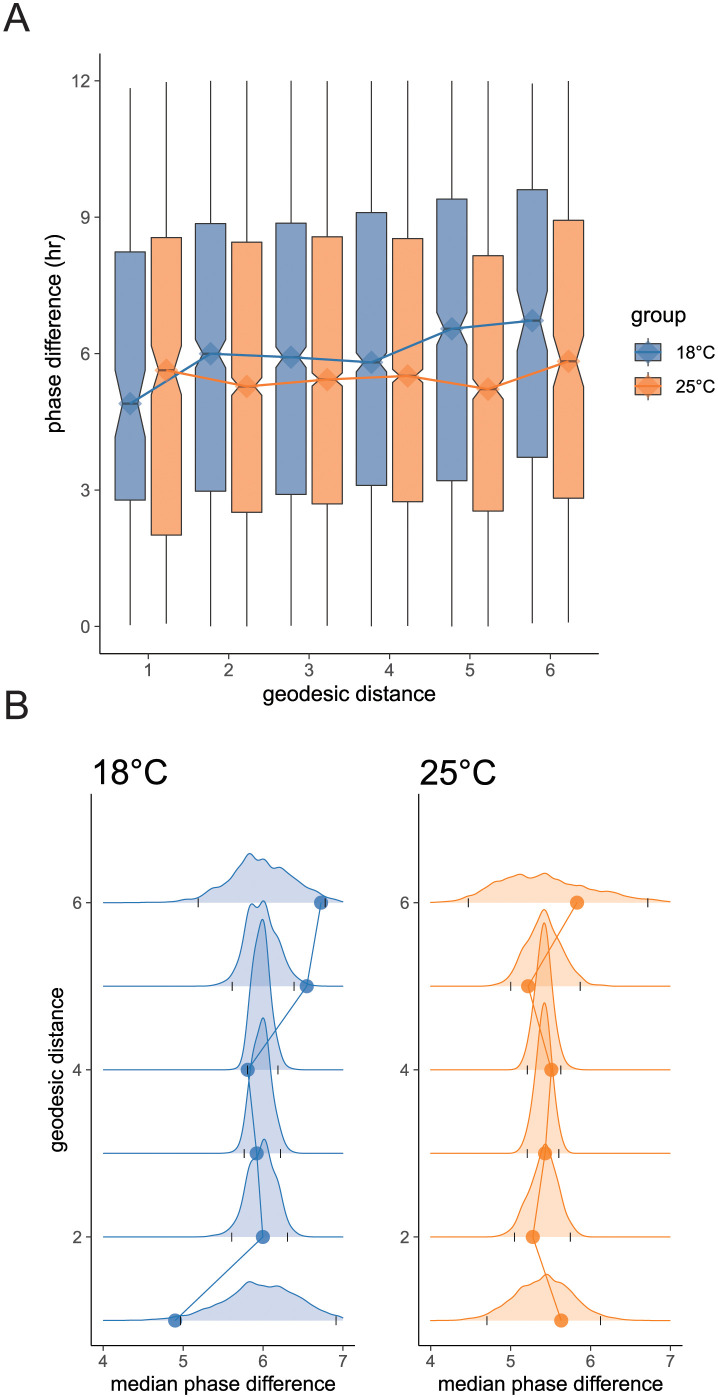
Phase differences with respect to the gene regulatory network. (A) Distribution of phase differences of gene pairs with at given geodesic distances. Lines connect the median under the two temperatures. (B) Observed median phase difference (points) at each geodesic distance compared to the null distribution (curves) of expected phase differences at each distance. Grey tick marks on the *x*-axis below each distribution indicate the 0.025 and 0.975 quantiles of the null distribution. Points lying outside the tick-marks are significant at the *α* = 0.05 level.

If it is the case that cycling genes are coordinated by the gene regulatory network, we may expect that genes that are closer together on the network will exhibit smaller phase differences than those that are farther apart. To test this idea, we quantified the association between phase differences between gene pairs and their geodesic distances (length of the shortest path on the network, illustrated in Fig 2C). As shown in Fig 4A, we found that the phase difference distributions computed at the two temperatures exhibit distinct patterns. At 25°C, the median phase difference at each geodesic distance remained approximately constant (slope = 0.025, *p* = 0.69, *df* = 4, linear regression). By contrast, at 18°C the median phase difference steadily increased with geodesic distance (slope = 0.30, *p* = 0.019, *df* = 4, linear regression). This implies that at 18°C, phases are organized with respect to the gene regulatory network. To determine if such organization would occur by chance, we constructed a null model by randomly reassigning the locations of cycling genes on the network 5000 times (Fig 4B). Compared to the null model, the phase differences of immediate neighbours at 18°C were significantly smaller than that of any pair of genes selected by chance (*p* = 0.018, Fig 4B). Likewise, we observed that the phase differences of gene pairs that are distant on the network tend to be large (*p* = 0.003 and *p* = 0.03 for geodesic distances of five and six respectively), consequent of the fact that genes with similar phases are nearby. At 25°C however, none of the median phase differences on the network were significantly larger or smaller than expected by chance.

We considered the association between network position and phase to be evidence of phase organization across the network. We observed this in the low temperature condition only, and we reason that there are several plausible explanations: either we have reduced ability to detect cycling genes and estimate phases at 25°C due to increased transcriptional noise or increased cell-cell desyncrony, or there is a true biological reprogramming of the circadian transcriptome. To investigate these possibilities, we conducted two additional analyses.

First, we reasoned that an higher temperatures may increase transcriptional noise, hindering our ability to detect cycling genes at 25°C compared to 18°C. To test this, we exploited the fact that most genes do not show rhythmic behavior, quantified the gene expression variance for all genes in all four datasets (two from V1 and two from V2), as shown in S7(A) Fig. We found that gene expression variance was higher at 25°C than 18°C in the V1 experiment (*p* = 0.0005, two tailed Wilcoxon signed rank test), but (strongly) higher at 18°C relative to 25°C in the V2 experiment (*p* = 2 × 10^−16^, two tailed Wilcoxon signed rank test). When the variances from V1 and V2 are combined, the increased variance at 18°C relative to 25°C in V2 dominates, such that 18°C appears to have higher variance overall (*p* = 2 × 10^−16^). This result, and the lack of consistent association between V1 and V2, suggests that there is no evidence for increased variance at higher temperatures.

Alternatively, we might also observe a loss of rhythmicity if cells become asynchronous under higher temperature, effectively damping the oscillations that could be observed in the bulk; because our data were collected using bulk RNA sequencing, successful detection of rhythmic activity requires most cells to oscillate synchronously. A loss of synchrony amongst cells would result in a lower amplitude observed in the bulk, even amongst cycling genes. We thus tested whether the amplitude of genes that were detected as cycling under both temperatures exhibited reduced amplitude at higher temperatures (S7(B) Fig). We observed decreased oscillation amplitude at 25°C relative to 18°C in the V1 experiment (*p* = 5 × 10^−5^, two tailed Wilcoxon signed rank test), but an increased oscillation amplitude at 25°C relative to 18°C in the V2 experiment (*p* = 0.017, two tailed Wilcoxon signed rank test). Combining the V1 and V2 amplitudes destroys any association between temperature and amplitude (*p* = 0.2). As before, we observed no consistent association between oscillation amplitude and temperature, implying that the tissue is likely to be equally well synchronized at 18°C and 25°C.

These results suggest that the effect we observe (increased cycling and phase organization at 18°C) is due to temperature–dependent changes in circadian gene regulation. By considering oscillation amplitude and gene expression variance, we reason that the lack of phase organization at 25°C is not simply a consequence of either increased transcriptional noise or loss of cell-cell synchronization.

## Discussion

By conducting bulk RNA sequencing at different temperatures, we identified genes in *Drosophila melanogaster* that cycle in a temperature–dependent manner. Genes under circadian control at 18°C tend to peak later in the morning than those oscillating at 25°C. We further found that the location of cycling genes on the gene regulatory network exhibits statistical evidence of phase assortativity (clustering of similar phases on the network) at 18°C, and thus hypothesize that rhythmic gene expression is coordinated along the network at lower temperatures. The differences in cycling primarily affected the activity of pathways associated with lipid metabolism, suggesting that the fruit-fly uses the circadian clock to alter its metabolism in lower temperature conditions.

Our study extends previous research in several ways. First, existing studies of circadian oscillations have focused primarily on the identification of rhythmic transcripts [7] or the function and impact of a small group of genes under circadian control [51, 52]. However, these studies have have not shed light on circadian oscillations in the context of gene-gene interactions. Our network-based approach provides additional insight into how the circadian rhythm coordinates gene expression. Second, our work demonstrates that a long–lasting temperature change, spanning multiple days, can alter circadian transcriptional regulation. This complements previous studies demonstrating that diurnal temperature cycles can also drive the cyclic expression of genes [14, 53, 54]. By assaying gene expression at constant temperatures, we isolate temperature–specific circadian oscillations from temperature–driven cycling. A more naturalistic experiment, in which a baseline change in temperature is combined with a diurnal thermal cycle about the new baseline, could be the focus of future work.

Our findings are the first to indicate that changes in circadian transcriptional control accompany non-cyclic changes in temperature. We propose that the fact that the morning gene expression peak shifts later at lower temperatures, consistent with a shorter winter day, may be evidence for a program of seasonal adaptation in the fly. That is, at lower temperatures, the fly not only changes its metabolic activity, it also changes *when* that metabolic activity happens. We emphasize here that the flies continued to receive a 12:12 light:dark photocycle, and thus this change was driven by temperature alone; transcriptomically, however, the fly appears to be anticipating a shorter photoperiod. The interplay between the clock and thermal acclimation has also been noted in the other direction; photoperiod cues have been shown to influence heat resistance in *Drosophila buzzatti* [55, 56]. While it has been shown that the circadian rhythm can act as a seasonal timer, with photoperiod cues altering behavioral adjustment to variations in daylength and season [57], our work indicates that temperature cues may enable the animal to *anticipate* changes in the photoperiod.

We further posit that the increased coordination of gene expression activity (i.e., the greater organization of cycling genes with respect to the network) may be advantageous for enhancing metabolic efficiency under suboptimal conditions (“suboptimal” in the sense that enzymatic activity will be reduced at 18°C due to Arrhenius scaling, and that *D. melanogaster* are observed to prefer 25°C [26, 55]). This conjecture is supported by other work [58] showing that circadian regulation of gene expression is necessary for flies to adapt to lower–nutrient conditions. In that study, flies given a 5% sucrose–yeast solution (vs. 15%, as in our study) exhibited lifespan elongation, but only when they had a functioning clock; *Clk* mutant flies, which lack behavioral and transcriptional rhythms, showed no change in lifespan as a function of dietary restriction. Clock coordination of gene expression (specifically, rhythmic control of proteolysis) was necessary to enable flies to live longer in nutrient–poor conditions. Similarly, flies at 18°C also exhibit lifespan elongation [59], and we propose that the clock may play a role in this as well by amplifying metabolic pathways at the times when the fly is likely to be most active (meteorological dawn and dusk). This hypothesis could be investigated by examining lifespan at different temperatures in wildtype and *Clk* mutant flies. As another possible follow-up experiment, one could attempt to induce particular genes in the network to oscillate using a GAL4-UAS system similar to Ulgherat et al [52]. By inducing certain genes to oscillate under direct control of the core clock, it may be possible to recreate the 18°C phase organization at 25°C. One may then study whether recovering the phase organization recapitulates phenotypes observed at 18°C, such as lifespan elongation [59].

Our observations suggest a mechanism by which having a clock may confer an evolutionary advantage. The “circadian resonance hypothesis” [60] postulates that synchronization between environment and oscillatory metabolic processes contributes to longevity, and multiple studies appear to support this idea [61–63]. *How* this occurs remains an open question, however. Our study suggests that rhythmic expression of metabolism–associated genes in the fat body and temperature–dependent phase organization may be conducive to circadian resonance throughout the year. Further insights into the fitness consequences of the interplay between the clock, metabolism, and temperature could be obtained by studying other *Drosophila* species. For instance, one may investigate whether species that exhibit lower cold resistance [26, 55, 64] also exhibit a smaller change in phase organization at low temperatures.

We acknowledge that our study only considers one type of environmental perturbation, namely a step change from high to low temperature. To fully understand how circadian control changes with environment, it would be useful to conduct similar studies under a wider range of conditions. For example, it would be valuable to conduct similar experiments at a range of temperatures in order to investigate how cycling and phase organization changes under finer temperature changes. It may also be of interest to investigate other tissues, as circadian synchronization and entrainment is known to differ between central and peripheral clocks [5, 65, 66]. Additionally, our study only probed transcriptomic changes; temperature–dependent post–translational modifications could not be probed here, but would give deeper insights into the effect of temperature on metabolism.

Finally, an open question is how quickly the genes change their cycling behavior to adapt when the temperature changes. Observing the transient dynamics of gene expression activity immediately following the temperature change can yield insights into this question. For investigations of this question using the V2 data, the reader is referred to [41].

## Materials and methods

### Experimental study design

All experiments were carried out with Iso31 flies (Bloomington Drosophila Stock Center ID: 5905). Age–matched female flies were collected and aged for ∼3 days with males before being transferred to an entrainment incubator. All flies were entrained with 12:12 LD cycles, with a 15% sucrose–yeast diet. Following three days of entrainment, the experimental group was shifted to 18°C (at approximately seven days old), while the control group was kept at 25°C.

For the V1 experiment, flies were held in 25°C or 18°C for five days prior to sampling. Fatbody samples were collected on Day5 from ZT02 to ZT24 every two hours with two replicates at each time point (Fig 1B).

For the V2 experiment, sampling began immediately following the temperature perturbation, with one replicate collected every two hours and another every six hours during the first two days(from ZT0 day one to ZT12 day two). Additional samples were then collected after the flies adapted to the new conditions, with one replicate collected every two hours and another replicate approximately every six hours from ZT04 Day 4 to ZT18 Day 5 (Fig 1B). In the present analysis, we used only the data collected on day four and five.

### Fat body dissection

Flies were directly dissected without dry ice to harvest fat tissues in the abdomen. Pinned flies were cut to remove organs in the abdomen (intestine, ovaries, malpighian tubules, etc.). Fat tissue attached to epidermis was collected. Fat body from ∼10 flies were harvested within 10 minutes for each time point of RNA-Seq analysis.

### RNA extraction

RNA was isolated from the abdominal fat bodies using Trizol LS (ThermoFisher, #10296028). 300 *μ*L of Trizol LS was added to fat bodies in 100 *μ*L of PBS. The tissue was homogenized with a motorized pestle for 2 minutes before adding another 600 *μ*L of Trizol LS (3:1 mixture of Trizol LS:PBS). The resulting solution was centrifuged at 12,000g for 10 minutes at 4°C. The aqueous supernatant layer was collected in a new tube, while carefully avoiding disturbing the other layers of the phase separated solution. RNA was extracted from the aqueous supernatant layer by vigorously shaking with 240 *μ*L of chloroform (Fisher Scientific, #C298), again carefully avoiding other layers following phase separation. The aqueous phase was transferred to a new tube and the RNA was precipitated by incubating at room temperature with 500*μ*L of 100% isopropanol (Sigma-Aldrich, #I9516). Following centrifugation at 12,000g for 10 minutes at 4°C, the supernatant was removed leaving only the RNA pellet. The pellet was washed with 1 mL of 75% ethanol (Sigma-Aldrich, #E7023), then air dried for 5–10 minutes before resuspension in RNase–free water.

### RNA-seq, experiment V1

cDNA libraries were constructed with poly(A) selected mRNA using Truseq RNA library preparation kit and then sequenced at the Genomics Core Facility at the University of Chicago on Illumina HiSeq 2000 System.

### RNA-seq, experiment V2

Purified RNA was sent to Novogene (Sacramento, CA) for library preparation. Libraries were prepared from mRNA purified from total RNA using poly-T oligo-attached magnetic beads (NEBNext Ultra II RNA Library Prep kit for Illumina, New England Biolabs, E7775). Non-stranded library preparation was carried out using the NEBNext Ultra II RNA Library Prep kit for Illumina according to manufacturer protocol. Libraries were subsequently sequenced on a Novaseq 6000 S4 flow cell. 20 million paired-end reads (PE150) were generated for each sample.

### Read alignment

Bulk RNA seq data were first quality checked using FastQC [30] and trimmed using Atropos [31]. Reads (deduplicated for V2) were aligned and quantified using STAR [32] and RSEM [33] to the Ensembl *Drosophila melanogaster* BDGP6.32 reference (release 107) using standard parameters (see S1 Appendix for detailed protocols).

### Data preprocessing

We selected genes with median TPM (transcript per million)> 5 in either the 18°C or 25°C condition, consistently across the two experiments. 6774 genes passed this filter in total. Only these genes were used in further analysis.

### Quantification of phase differences

The angular separation between two angles *φ*_1_ and *φ*_2_ in radians is given by

$$\begin{array}{c} \Delta \varphi =\text{arg}\;(\frac{e^{i\varphi_{1}}}{e^{i\varphi_{2}}})\;. \end{array}$$ (1)

In hours, the signed phase difference Δ*ϕ* is thus

$$\begin{array}{c} \Delta \phi =\text{arg}\;(\frac{\text{exp}\;(i\frac{2\pi }{24}\phi_{1})}{\text{exp}\;(i\frac{2\pi }{24}\phi_{2})})\frac{24}{2\pi }\;, \end{array}$$ (2)

where *ϕ*_1_ and *ϕ*_2_ are the phases (in hours) of detected cycling genes. Δ*ϕ* > 0 indicates a *phase advance* of *ϕ*_1_ relative to *ϕ*_2_; Δ*ϕ* < 0 indicates a *phase delay*.

The absolute phase difference |Δ*ϕ*| in hours is equivalent to

$$\begin{array}{c} \left|\Delta \phi \right|=\text{min}\;(|\phi_{1}-\phi_{2}|,24-|\phi_{1}-\phi_{2}|)\;. \end{array}$$ (3)

### Identification of rhythmic genes

Harmonic regression was used to identify cycling genes and estimate phases. For each gene, we have two *p* values and phase estimates, one from the V1 experiment and one from the V2 experiment. A gene is considered to be cycling if both the *p* < 0.1 in *both* V1 and V2, and the phase estimates differ by no more than three hours (|Δ*ϕ*| < 3*h*).

Although we used harmonic regression to conduct cycling detection, which can be vulnerable to false positives, we reasoned that our combination of the V1 and V2 data mitigated this concern. Additionally, we compared the performance of harmonic regression and JTK-CYCLE and observed that while *p*-values from JTK-cycle tend to be higher, the two methods produce the same phases and correlated *p*-values (see S1 Fig).

### Differential cycling analysis

Differential cycling analyses were conducted using the limorhyde [34] and the limorhyde2 [35] package in R following its standard procedures with default parameters.

### Network analysis

The network used in our analysis was constructed using the graphite package in R [36, 37]. We first constructed a graph of all genes by taking the graph union of all pathways from the Reactome [38] database (as given in the graphite R package). To facilitate later analysis, the largest connected component within this graph of all genes was extracted. Given our interest in understanding phase organization, small disconnected networks will not be as informative, hence this largest component was used for subsequent analysis. We note, furthermore, that the largest connected component comprises the majority of genes and edges in the graph. The original network contained 4080 nodes and 212,808 edges; the largest connected component contained 3875 nodes and 205,758 edges.

### Over-representation analysis

Over-representation analysis of individual Reactome pathways was conducted using “enrichPathway” function in clusterProfiler package in R [39, 40] with default parameters. For GO analysis, we used the “enrichGO” function in the same package with the same default parameters. All genes that passed the median threshold were used as the universe input.

## Supporting information

S1 Fig Comparison of harmonic regression and JTK-cycle. (A) Phases of cycling genes estimated using harmonic regression and JTK-CYCLE. (B) *p*-values estimated using harmonic regression and JTK-CYCLE (log scale). In all panels, the red line indicates *y* = *x*.
(PDF)

S2 Fig Idntifying circadian genes. Heatmap showing the *Z*-scored TPM of genes identified as cycling in the V2 experiment. For visualization purposes, replicates were averaged.
(PDF)

S3 Fig Computing false discovery rate. Number of identified cycling genes (A) and false discovery rate (B) as a function of harmonic regression *p*-value thresholds under different |Δ*ϕ*| thresholds. Blue: 25°C. Black: 18°C. Our selected thresholds, *p* < 0.1 and |Δ*ϕ*| < 3 yields FDRs of 0.07 and 0.047 in 18°C and 25°C, respectively.
(PDF)

S4 Fig The core clock genes showed robust oscillation across experiments. (A) Phases of the core clock genes estimated in the V1 and V2 experiments. The dashed line indicates *y* = *x*. (B) Centered TPM of core clock genes. Replicates were concatenated for V1 and averaged for V2 for visualization.
(PDF)

S5 Fig Estimating phase shift across experiments. Phase shift of genes that cycle under both temperatures, as estimated via limorhyde2 for the V2 and V1 experiments. The red line indicates *y* = *x*.
(PDF)

S6 Fig Network properties of cycling genes. (A) Degree distributions of genes detected as cycling under the two temperatures, as well as all genes. (B) Geodesic (network) distance distributions for gene pairs that are cycling under the two temperatures, as well as all gene pairs.
(PDF)

S7 Fig Comparison of variance and amplitude in 25°C relative to 18°C in the two datasets. (A) Gene expression variance for all genes passing filtration in V1 and V2. (B) Oscillation amplitude of genes cycling under both temperatures in V1 and V2. In all plots, red lines indicate *y* = *x*.
(PDF)

S1 Appendix Supplementary information. Additional details regarding read alignment and the application of JTK-CYCLE.
(PDF)

S1 Table Information on common cyclers across experients. Harmonic regression phase, *p* value, and limorhyde *p* value of genes that were considered cycling in both experiments.
(CSV)

S2 Table Functional analysis of 25°C cyclers. Reactome pathways enriched by genes considered to be cycling under 25°C.
(CSV)

S3 Table Functional analysis of 18°C cyclers. Reactome pathways enriched by genes considered to be cycling under 18°C.
(CSV)

S4 Table Functional analysis of common cyclers. Reactome pathways enriched by genes considered to be cycling under both temperatures.
(CSV)
