# Supplementary figures and images for: Temperature-driven coordination of circadian transcriptional regulation
Source: PLoS Comput Biol. 2024 Apr 22;20(4):e1012029. doi: 10.1371/journal.pcbi.1012029 (PMC11108206; doi:10.1371/journal.pcbi.1012029)

A

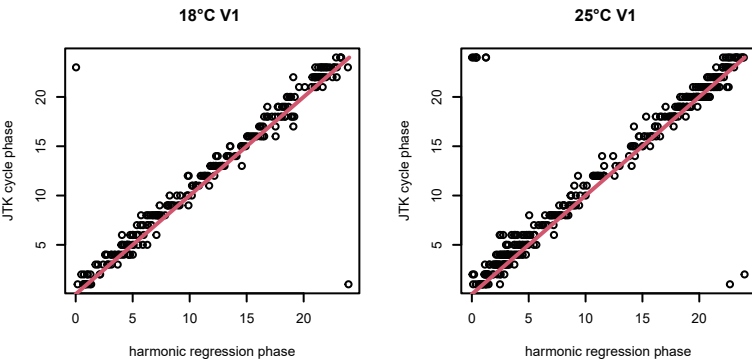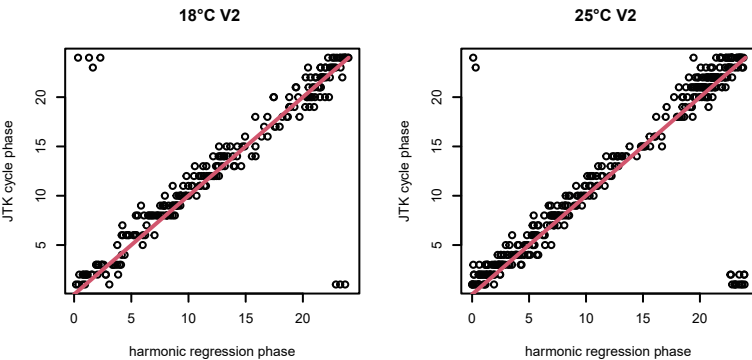

B

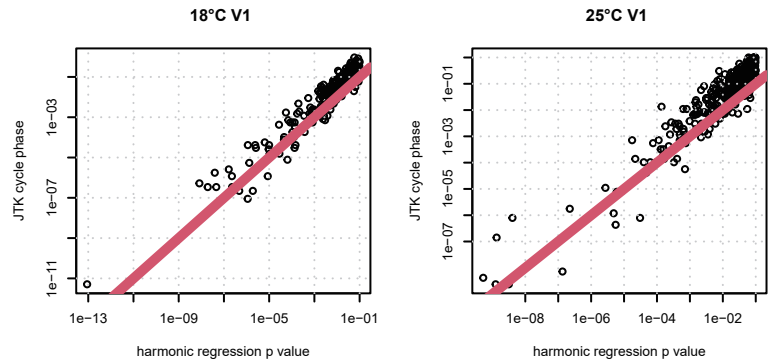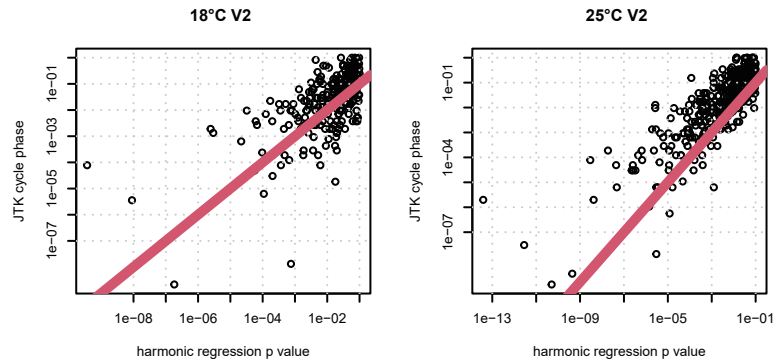

Supplement: S1 Fig — (A) Phases of cycling genes estimated using harmonic regression and JTK-CYCLE. (B) p-values estimated using harmonic regression and JTK-CYCLE (log scale). In all panels, the red line indicates y = x. (PDF) [file pcbi.1012029.s001.pdf]

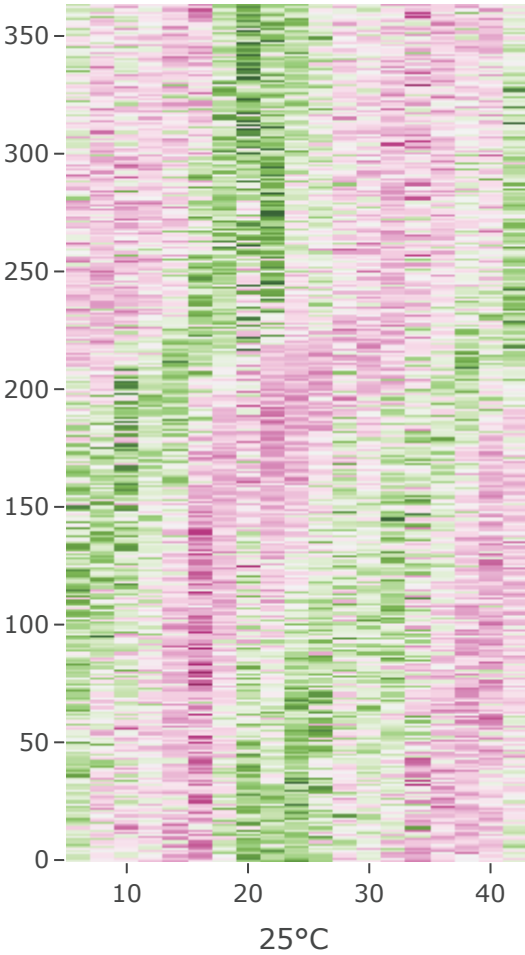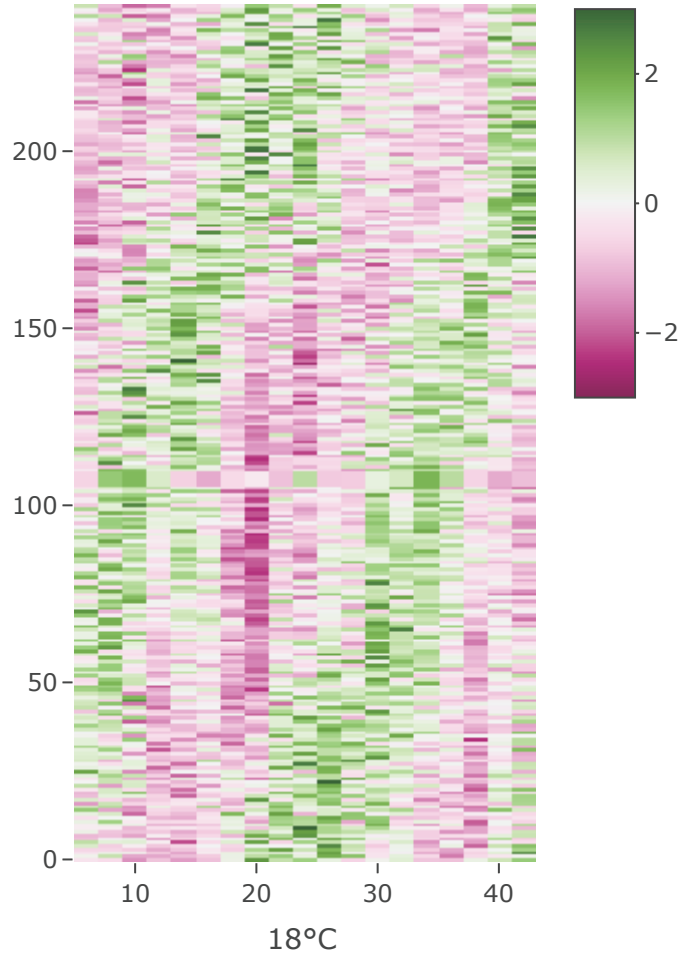

Supplement: S2 Fig — Heatmap showing the Z-scored TPM of genes identified as cycling in the V2 experiment. For visualization purposes, replicates were averaged. (PDF) [file pcbi.1012029.s002.pdf]

A

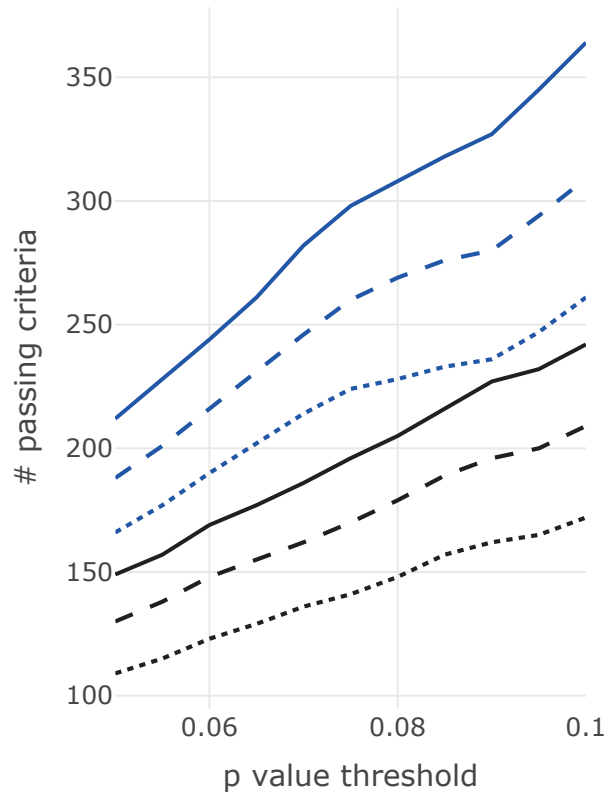

B

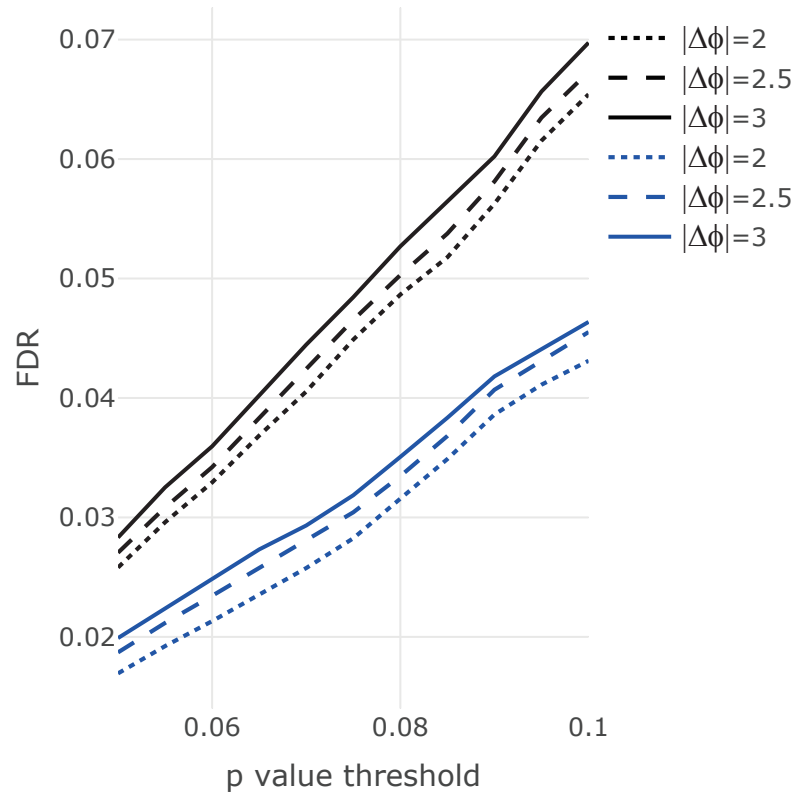

Supplement: S3 Fig — Number of identified cycling genes (A) and false discovery rate (B) as a function of harmonic regression p-value thresholds under different |Δϕ| thresholds. Blue: 25°C. Black: 18°C. Our selected thresholds, p < 0.1 and |Δϕ| < 3 yields FDRs of 0.07 and 0.047 in 18°C and 25°C, respectively. (PDF) [file pcbi.1012029.s003.pdf]

A

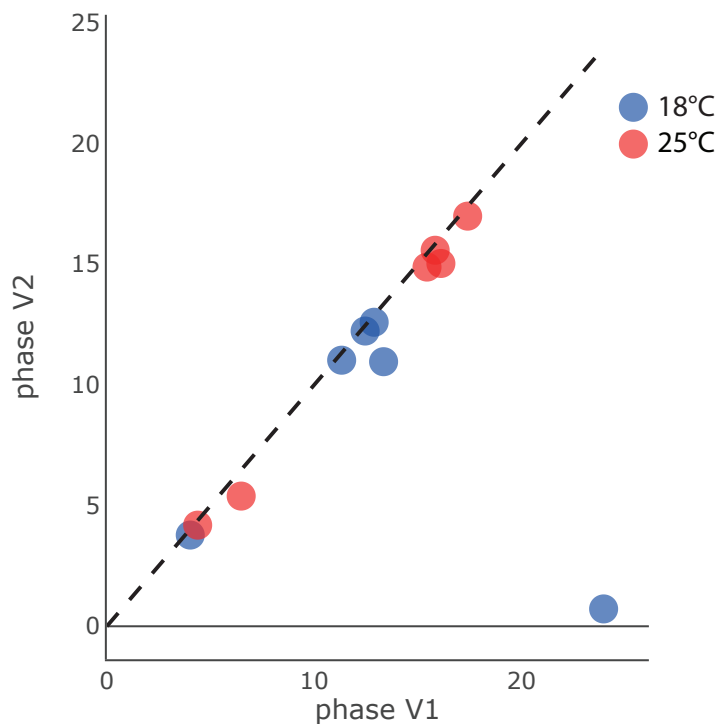

B

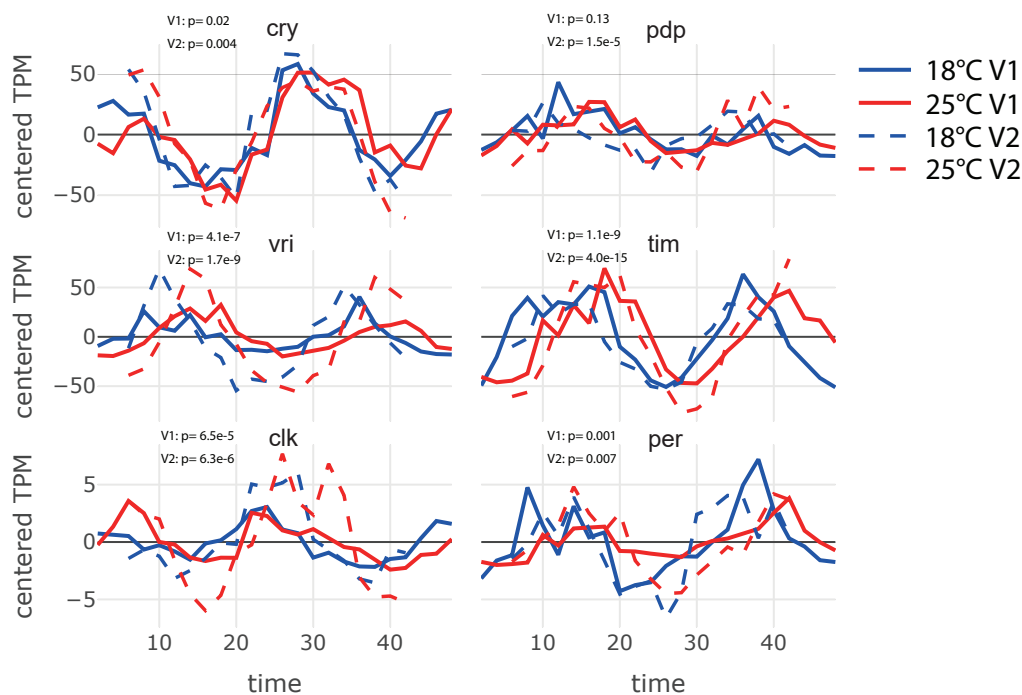

Supplement: S4 Fig — (A) Phases of the core clock genes estimated in the V1 and V2 experiments. The dashed line indicates y = x. (B) Centered TPM of core clock genes. Replicates were concatenated for V1 and averaged for V2 for visualization. (PDF) [file pcbi.1012029.s004.pdf]

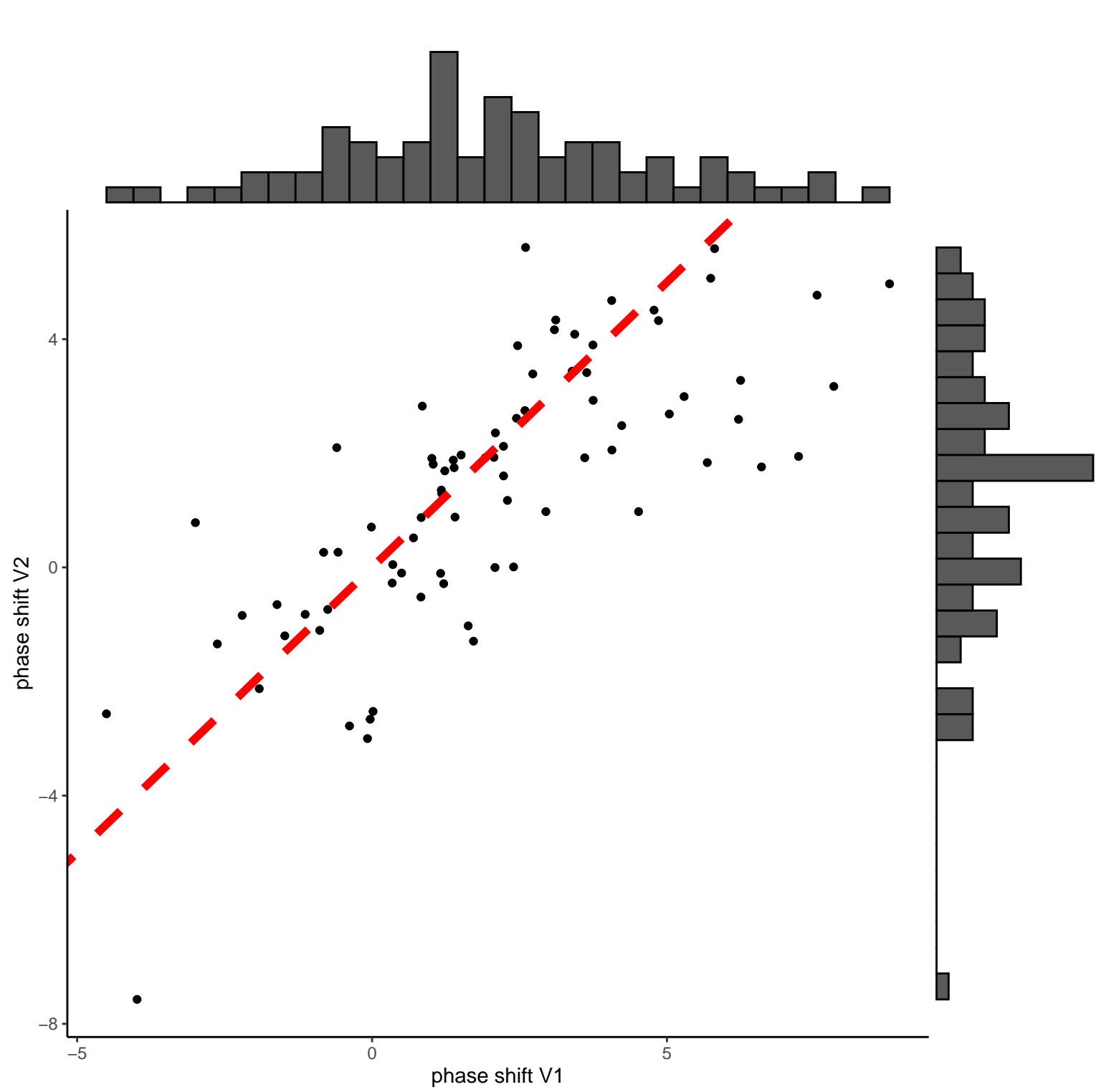

Supplement: S5 Fig — Phase shift of genes that cycle under both temperatures, as estimated via limorhyde2 for the V2 and V1 experiments. The red line indicates y = x. (PDF) [file pcbi.1012029.s005.pdf]

A

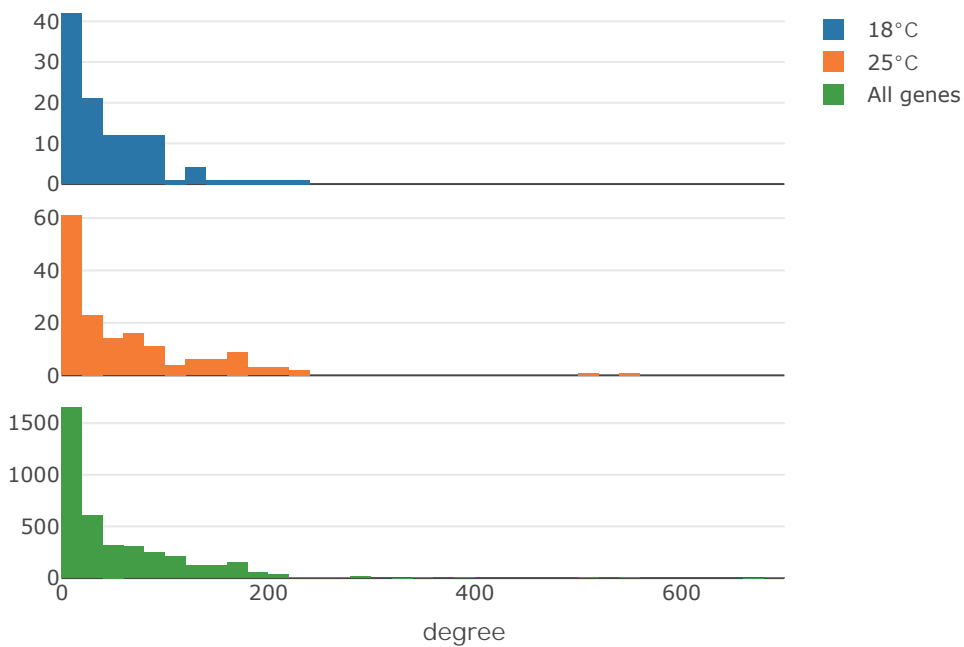

B

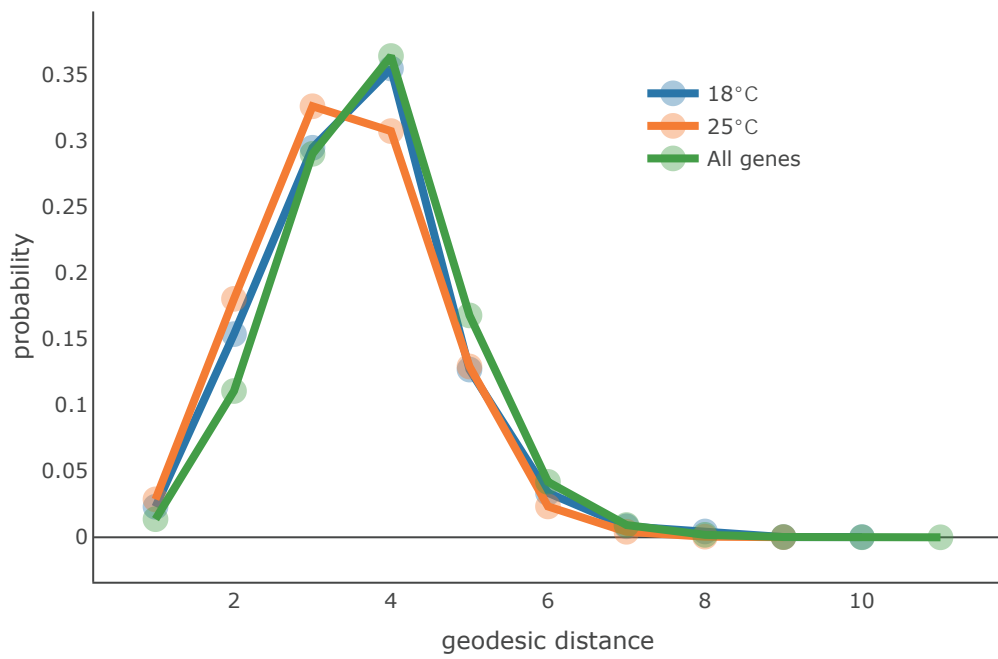

Supplement: S6 Fig — (A) Degree distributions of genes detected as cycling under the two temperatures, as well as all genes. (B) Geodesic (network) distance distributions for gene pairs that are cycling under the two temperatures, as well as all gene pairs. (PDF) [file pcbi.1012029.s006.pdf]

**A****V1**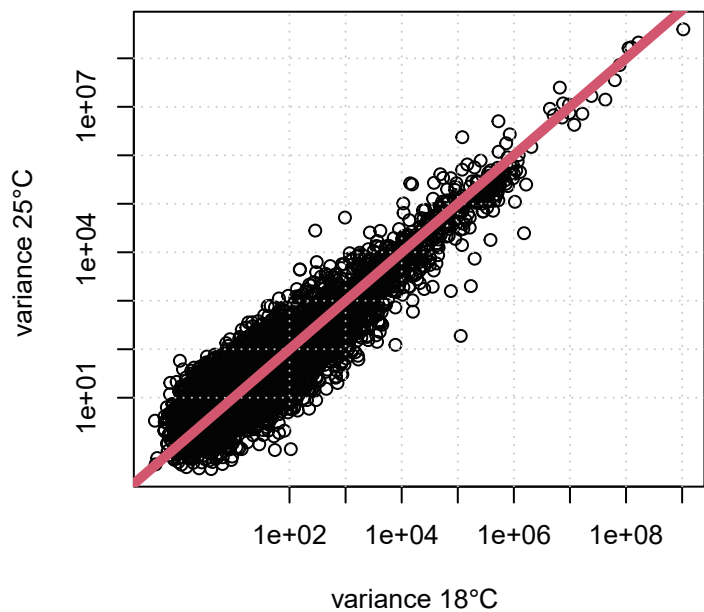**V2**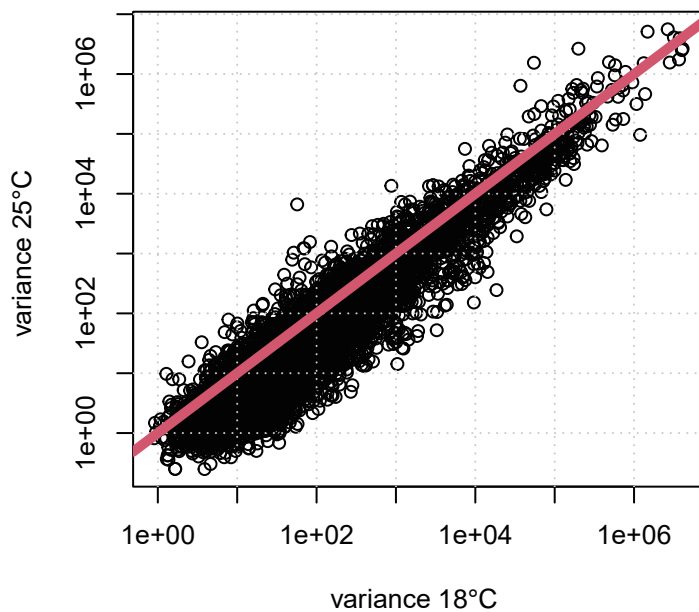**B****V1**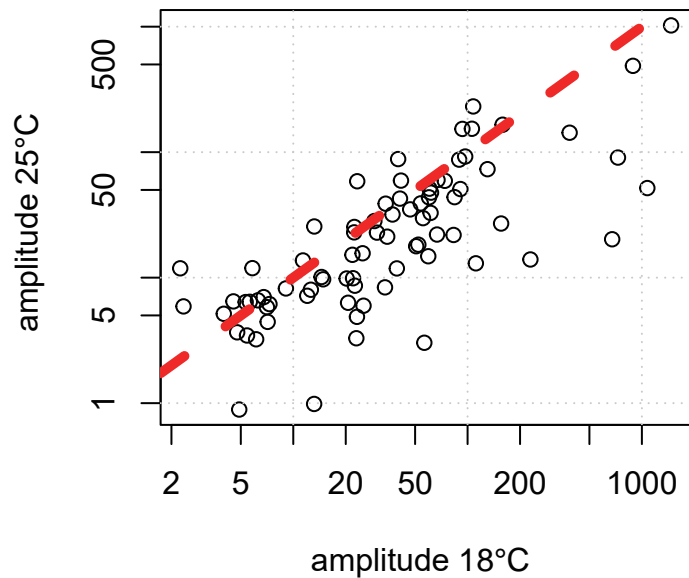**V2**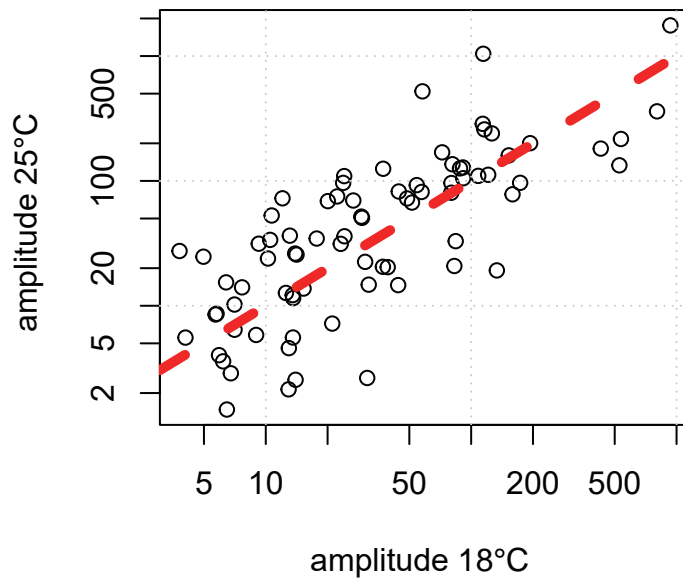

Supplement: S7 Fig — (A) Gene expression variance for all genes passing filtration in V1 and V2. (B) Oscillation amplitude of genes cycling under both temperatures in V1 and V2. In all plots, red lines indicate y = x. (PDF) [file pcbi.1012029.s007.pdf]
