# Supplementary material for: Temperature-driven coordination of circadian transcriptional regulation
Source: PLoS Comput Biol. 2024 Apr 22;20(4):e1012029. doi: 10.1371/journal.pcbi.1012029 (PMC11108206; doi:10.1371/journal.pcbi.1012029)
Supplement: S1 Appendix — Additional details regarding read alignment and the application of JTK-CYCLE. (PDF) [file pcbi.1012029.s008.pdf]

Supplementary Material

Bingxian Xu[1,2], Dae-Sung Hwangbo[3,4], Sumit Saurabh[5], Clark Rosensweig[2,4], Ravi Allada[2,4,10,11], William L. Kath[1,2,4,8], and Rosemary Braun[1,2,6,7,8,9]

[1]Department of Molecular Biosciences, Northwestern University, Evanston, Illinois, United States of America
[2]NSF-Simons Center for Quantitative Biology, Northwestern University, Evanston, Illinois, United States of America
[3]Department of Biology, University of Louisville, Louisville, Kentucky, United States of America
[4]Department of Neurobiology, Northwestern University, Evanston, Illinois, United States of America
[5]Department of Biology, Loyola University, Chicago, Illinois, United States of America
[6]Department of Engineering Sciences and Applied Mathematics, Northwestern University, Evanston, Illinois, United States of America
[7]Department of Physics and Astronomy, Northwestern University, Evanston, Illinois, United States of America
[8]Northwestern Institute on Complex Systems, Northwestern University, Evanston, Illinois, United States of America
[9]Santa Fe Institute, Santa Fe, New Mexico, United States of America
[10]Michigan Neuroscience Institute, University of Michigan, Ann Arbor, Michigan, United States of America
[11]Department of Anesthesiology, University of Michigan, Ann Arbor, Michigan, United States of America

April 6, 2024

Read alignment

Basic quality checking of sequence files was performed with FastQC [1]. Paired-end reads were first trimmed using Atropos version 1.1.31 [2] using the options

```
atropos trim --aligner insert -a AGATCGGAAGAGCACACGTCTGAACTCCAGTCA \
       -A AGATCGGAAGAGCGTCGTGTAGGGAAAGAGTGT --minimum-length 50
```

Reads were then aligned and quantified using STAR [3] (version STAR_2.7.10a_alpha_220818) and RSEM [4] (version 1.3.1). STAR and RSEM indexes were first built using the Ensembl *Drosophila melanogaster* BDGP6.32 reference (release 107) using standard parameters. STAR was used with the options

```
--outFilterType BySJout --alignIntronMax 1000000 \
--quantMode GeneCounts TranscriptomeSAM
```

to produce raw counts and also a BAM file with reads aligned to transcriptome. RSEM was then used with options `--paired-end --strandedness none` to produce tags-per-million (TPM) counts for each gene from transcriptome alignments. Postprocessing of the count data into table form was performed with custom Perl, Python and shell scripts.

Because FastQC reported significant sequence duplication in the samples, we also performed the same analysis as above after deduplicating the reads. First, a BAM file was created using STAR with the options

```
--outFilterType BySJout --alignIntronMax 1000000 \
--outSAMmultNmax 1 --outSAMtype BAM SortedByCoordinate
```

to produce only uniquely mapped reads, and then duplicate paired-end reads were removed using bamUtil [5] with the options

```
bam dedup --rmDups --excludeFlags 0xB04 --oneChrom
```

The resulting reads were then re-aligned with STAR to produce a BAM file with reads aligned to the transcriptome, followed by RSEM with the same options as above to produce TPM counts. De-duplication significantly decreased the number of reads with large TPM values (e.g., > 100), and as the result the TPM values of genes with smaller TPM values were increased by approximately a factor of 1.6-1.7. Deduplicated reads were used throughout the paper.

Single-end reads were quality-assessed with FastQC and then trimmed with Atropos using the options

```
atropos -a AGATCGGAAGAGCACACGTCTGAACTCCAGTCAC --minimum-length 16
```

Reads were then aligned and quantified using STAR with the same options as before,

```
--outFilterType BySJout --alignIntronMax 1000000 \
--quantMode GeneCounts TranscriptomeSAM
```

followed by RSEM with the options `--strandedness none`.

Comparison against JTK-CYCLE

JTK-CYCLE was used to validate the performance of harmonic regression. For the V2 experiment, replicates were averaged first before the standard JTK-CYCLE protocol with period 24 hr and sampling interval 2 hrs. p values computed from JTK-CYCLE were highly correlated with those computed using harmonic regression (log linear regression: V1 25°C slope: 0.81; V1 18°C slope: 0.85; V2 25°C slope: 0.69; V2 18°C slope: 0.80; S1B Fig).

References

[1] S. Andrews. FastQC: a quality control tool for high throughput sequence data. Available online at https://www.bioinformatics.babraham.ac.uk/projects/fastqc/, 2010. Accessed 7 November 2022.

[2] John P. Didion, Marcel Martin, and Francis S. Collins. Atropos: specific, sensitive, and speedy trimming of sequencing reads. *PeerJ*, 5:e3720, 2017.

[3] Alexander Dobin, Carrie A. Davis, Felix Schlesinger, Jorg Drenkow, Chris Zaleski, Sonali Jha, Philippe Batut, Mark Chaisson, and Thomas R. Gingeras. STAR: ultrafast universal RNA-seq aligner. *Bioinformatics*, 29(1):15–21, January 2013.

[4] Bo Li and Colin N. Dewey. RSEM: accurate transcript quantification from RNA-Seq data with or without a reference genome. *BMC Bioinformatics*, 12:323, 2011.

[5] Bamutil. https://genome.sph.umich.edu/wiki/BamUtil, 2011. Accessed 7 November 2022.
